# Para-Cycling Performance was Rather Limited by Physiological than Functional Factors

**DOI:** 10.3389/fphys.2012.00327

**Published:** 2012-08-15

**Authors:** Pierre-Marie Leprêtre, Thierry Weissland, Jean Slawinski, Philippe Lopes

**Affiliations:** ^1^Laboratoire de Recherche Adaptations Physiologiques à l’Exercice et Réadaptation à l’Effort, Université de Picardie Jules VerneAmiens, France; ^2^Centre de Recherches Sur le Sport et le Mouvement, Université Paris OuestNanterre, France; ^3^Equipe de Recherche Dégénérescence et Plasticité Neuromusculaire, Centre d’Études de la Sensorimotricité, UMR-8194, LNRS CNRS, Université Paris DescartesParis, France

The purpose of the Para-cycling classification is to minimize the impact of impairment on the outcome of competition, so that an athlete’s success in competition relies on training, physical fitness, and personal athletic talent (UCI Cycling Regulations, 2011). Athlete evaluation is done in compliance with the International Paralympic Committee (IPC) Classification Code and International Standard on Athlete Evaluation. A classification panel for athletes with physical impairments in Handbike, Tricycle, and Cycle consists of three International Cycling Union (UCI) accredited classifiers: a medical doctor, physiotherapist, and sports technician. Athletes are classified according to the extent of activity limitation resulting from their impairment. This places athletes according to how much their impairment affects core determinants of performance in cycling. The chapter V of UCI Para-cycling classification guide (UCI Cycling Regulations, 2011) stipulated that the “functional ability of the athletes will decide the final classification” which depends on the nature of spinal cord lesion (complete or incomplete), neurological impairment or amputation. A classification scale is used to include athletes suffering from different pathologies (neurological, amputation) but having comparable multiple functional impairments in the same race category. Since 2010, the UCI included two impairments in the cycling classification: neurological-impairments, with central or peripheral damage, and orthopedic impairments. For example in C1 class, the athletes with hemiplegia, diplegia, athetosis, or ataxia with single above knee and arm amputation.

Additionally, a corrective factor essentially based on time – speed relationship is used during Paralympic games “for equity and to be able to compare the podium athlete/medal contenders” (http://www.uci.ch/Modules/BUILTIN/getObject.asp?MenuId=MTI1NjA&ObjTypeCode=FILE&type=FILE&id=NjY3OTE&LangId=1). If the goal of the corrective tool is to ensure that the three athletes on the podium are the best ones, this raised the question why the classification takes into account only the functional factors with the application of a time coefficient in more severely disabled sport classes. Based on the literature (Crouse et al., 1990; Hutzler et al., 1998; Sezer et al., 2004; Ward-Smith, 1985) and our comparison of intergroup performance established in different categories during the last world para-cycling championships (Table 1), it seems the severity of the athlete’s disability is associated with a reduced performance, whatever the duration of effort. One exception was the cycling performance occurred on individual road time trial (TT) and the 1-km Standing Start (1-km Individual TT, i.e., 1-km ITT) events for C4 and C5 categories. This result would reflect, in part, the difference in upper limb response to heterogeneous impairments among C4–C5′ athletes and, therefore, a difference in their gross efficiency (GE), i.e., the ratio between work and energy (Janssen et al., 2001; Leirdal and Ettema, 2011; Stone and Hull, 1993). Previous findings (Crouse et al., 1990; Janssen et al., 2001; Hutzler et al., 1998; Sezer et al., 2004) already suggested that reduced exercise tolerance in amputee and neurological subjects may be due to their reduced GE compared to healthy counterparts. Functional differences in balance, muscle, and motor control between amputees and cerebral palsy suggested that GE and therefore cycling performance are not similar. In a bilateral above knee amputee, Crouse et al. (1990) reported that walking with prostheses requires significant increase in energy expenditure and induces a shorter time to exhaustion compared to controls. Primary effects of stroke on sensorimotor function also include sensory-perceptual dysfunction which could induce technical impairment and a shorter time to exhaustion. However, no significant relationship was found between time to fatigue and motor disability (Corbett, 2009). Sezer et al. (2004) also showed a significant respiratory dysfunction in hemiplegic which could in part explain the difference in time to exhaustion compared with healthy subjects. It has been reported, in valid trained subjects, that maximal oxygen uptake (VO_2_max) rather than GE explained cycling performance (Capelli et al., 1998; Coyle, 1995). Taken together these previous results suggested that energy expenditure and aerobic capacity rather than motor ability may explain and contribute to cycling performance. During arm-cranking tests, Hutzler et al. (1998) also observed significant differences in aerobic capacity between athletes with different types of impairment (paraplegia, polio, or amputations). They showed that classification accounted for 30 and 38% of the variance in aerobic and anaerobic powers, respectively. This impact of disability was also significant on leg performance (Table 1).

**Table 1:** **Lap time (in seconds) to realize the best performance (±SD) of 1–km standing start for C1–C3 categories and the interclass time difference (delta, s) measured during the UCI World Para-cycling Track championships in 2011**.

Category	0–250 m	250–500 m	0–500 m (first 500 m)	500–750 m	250–750 m (middle 500 m)	750 m to 1 km	500 m to 1 km (Final 500 m)
C1*	25.5 (1.7)	18.2 (0.5)	43.7	18.9 (0.7)	37.1	20.3 (0.8)	39.2
C2*	25.4 (1.0)	17.4 (0.3)	42.8	17.5 (0.3)	34.9	18.2 (0.6)	35.7
C3	21.7 (0.7)	16.3 (0.5)	38.0	16.9 (0.4)	33.2	17.9 (0.6)	34.8
Delta	0–250 m	250–500 m	0–500 m (first 500 m)	500–750 m	250–750 m (middle 500 m)	750 m to 1 km	500 m to 1 km (Final 500 m)
C1–C2	−0.3	0.8	0.4	1.4	2.2	2.1	3.5
C2–C3	3.7	1.2	4.9	0.7	1.8	0.3	1.0
C1–C3	3.4	1.9	5.3	2.1	2.7	2.4	4.5

The performance corresponds to the average speed for the gold, silver, and bronze medalists (Ward-Smith, 1985; Katz and Katz, 1999). *New world record established during the race.

Intermediate times measured during 1-km ITT revealed similar pacing-profiles which were characterized by an initial acceleration followed by a progressive decay in split times (Table 1). Like in valid cyclists, the first 250 m split time was a primary determinant of total 1-km ITT time in para-cyclists. It seems almost certain that the initial acceleration property is limited by the disability level. The 2.4 s separating first and second C1 cyclists after the first 250 m also demonstrated that the nature of disorder (central or peripheral) impacted on performance. Over the remainder of the race, the split time difference between C1–C2 and C1–C3 continuously increased over time whereas it blunted between C2 and C3 athletes. These differences in intergroup responses could be attributable in part to (1) the difference in energy cost between C1 and C2–C3 categories (Capelli et al., 1998) and (2) the adopted pacing strategies. C3 were characterized by a quicker start compared to C2 cyclists. Previously, Corbett (2009) showed that an overly quick start was associated with a concomitant slowing toward the finish. Performance appears to be attributable to faster VO_2_ responses without a significant change in anaerobic contribution (Bishop et al., 2002). A faster start could also alter the metabolic responses and explain the inability to maintain exercise intensity. Energy requirements may elucidate in part the decrease in time difference from 250 m to 1-km between C2 and C3 and the similar performance between 1-km ITT and 3-km individual pursuit in C2 category during the 2011 world track para-cycling championship (Table 1). The absence of comparative studies on physiological responses between infraclass cyclists makes the task difficult for the IPC to deliver a combined title for the C1–C5 categories during Paralympic Games. Therefore, the question of equity and nation’s strategies remains during a unique race with different final ranking. Based on the metabolic model, cycling performance would be primarily determined by the value of oxygen uptake at the lactate threshold (VO_2-LT_) and its upper-limit (VO_2_max; Coyle, 1995; Capelli et al., 1998). Hence, higher VO_2_max values will increase the time spent at high intensities for less impaired athletes with higher chances of winning. Moreover, the decrease in time difference over the time would promote C2–C3 categories. Finally, the difference in physiological and residual muscle strength between amputated and spinal cord injuries athletes require detailed biomechanical and physiological investigations in order to measure precisely the impact of disability on cycling performance.

## References

[B1] Bishop D. Bonetti D. Dawson B. (2002). The influence of pacing strategy on VO2 and supramaximal kayak performance. Med. Sci. Sports Exerc. 34, 1041–1047 10.1097/00005768-200206000-00022 12048335

[B2] Capelli C. Schena F. Zamparo P. Monte A. D. Faina M. di Prampero P. E. (1998). Energetics of best performances in track cycling. Med. Sci. Sports Exerc. 30, 614–624 10.1097/00005768-199804000-00021 9565945

[B3] Corbett J. (2009). An analysis of the pacing strategies adopted by elite athletes during track cycling. Int. J. Sports Physiol. Perform. 4, 195–205 19567923 10.1123/ijspp.4.2.195

[B4] Coyle E. F. (1995). Integration of the physiological factors determining endurance performance ability. Exerc. Sport Sci. Rev. 23, 25–63 10.1249/00003677-199500230-00004 7556353

[B5] Crouse S. F. Lessard C. S. Rhodes J. Lowe R. C. (1990). Oxygen consumption and cardiac response of short-leg and long-leg prosthetic ambulation in a patient with bilateral above-knee amputation: comparisons with able-bodied men. Arch. Phys. Med. Rehabil. 71, 313–317 2327883

[B6] Hutzler Y. Ochana S. Bolotin R. Kalina E. (1998). Aerobic and anaerobic arm-cranking power outputs of males with lower limb impairments: relationship with sport participation intensity, age, impairment and functional classification. Spinal Cord 36, 205–212 10.1038/sj.sc.3100627 9554023

[B7] Janssen T. W. Dallmeijer A. J. van der Woude L. H. (2001). Physical capacity and race performance of handcycle users. J. Rehabil. Res. Dev. 38, 33–40 11322469

[B8] Katz J. S. Katz L. (1999). Power laws and athletic performance. J. Sports Sci. 17, 467–476 10.1080/026404199365777 10404495

[B9] Leirdal S. Ettema G. (2011). Pedaling technique and energy cost in cycling. Med. Sci. Sports Exerc. 43, 701–705 10.1249/MSS.0b013e3181f6b7ea 20798659

[B10] Sezer N. Ordu N. K. Sutbeyaz S. T. Koseoglu B. F. (2004). Cardiopulmonary and metabolic responses to maximum exercise and aerobic capacity in hemiplegic patients. Funct. Neurol. 19, 233–238 15776791

[B11] Stone C. Hull M. L. (1993). Rider/bicycle interaction loads during standing treadmill cycling. J. Appl. Biomech. 9, 202–218

[B12] UCI Cycling Regulations (2011). “Para-cycling sport class profiles,” in Para-Cycling, Part 16, Chap. V.

[B13] Ward-Smith A. J. (1985). A mathematical theory of running, based on the first law of thermodynamics, and its application to the performance of world-class athletes. J. Biomech. 18, 337–349 10.1016/0021-9290(85)90290-8 4008504

